# Effects of Alloying Elements and Mechanical Alloying on Characteristics of WVTaTiCr Refractory High-Entropy Alloys

**DOI:** 10.3390/ma16186194

**Published:** 2023-09-13

**Authors:** Chun-Liang Chen, Jyun-Hong Lin

**Affiliations:** Department of Materials Science and Engineering, National Dong Hwa University, Hualien 97401, Taiwan

**Keywords:** mechanical alloying, refractory metal, high-entropy alloy, phase formation

## Abstract

Refractory high-entropy alloys (RHEAs) are among the promising candidates for the design of structural materials in advanced nuclear energy systems. The effects of Cr, V, Ta, and Ti elements and ball milling on the microstructural evolution and mechanical properties of model RHEAs were investigated. The results show that W-rich BCC1 and Ta-rich BCC2 solid solution phases were generated after a long milling duration. After high-temperature sintering, the (Cr, Ta)-rich phase associated with the Laves phase was observed in the Cr-containing model RHEAs. In addition, a high level of Ti, Ta, and V contents promoted the in situ formation of oxide particles in the alloys. Complex TiTa_2_O_7_ and Ta_2_VO_6_ oxide phases were identified by TEM, which suggests a solid-state reaction of Ti-O, Ta-O, and V-O subjected to high-energy ball milling. The oxide particles are uniformly dispersed in the BCC matrix, which can result in dispersion strengthening and the enhancement of mechanical properties.

## 1. Introduction

In the past decade, several W-based binary alloys have been studied for nuclear structural materials, such as W-Ta, W-V, W-Ti, and W-Cr [1,2,3,4]. However, some W-based binary alloys exhibited brittle failure characteristics [5]. Refractory high-entropy alloys (RHEAs) are considered novel promising high-temperature materials for nuclear fusion applications [6,7,8]. Critical issues for the design of advanced nuclear reactors should be respected in regard to the effects of higher neutron flux, corrosive resistance, and higher operating temperatures [9]. RHEAs exhibit superior radiation resistance and thus have considerable potential for the development of nuclear structural materials. This is because of the unique properties of the severe lattice distortion and sluggish diffusion of RHEAs, which can lower the density of dislocation loops and offer less radiation-induced segregation [10,11,12,13].

Refractory metal elements with low activation are beneficial for the design of nuclear structural materials. It has been reported that the W, Ta, Ti, V, and Cr elements have low neutron activation properties and are suitable for the development of a new RHEA for fusion plasma-facing materials [14,15]. WTaTiVCr high-entropy alloys with enhanced physical and mechanical characteristics can be achieved by a powder metallurgical technique [15]. The low-activation TiVNbTa alloy has also been proposed as a high-temperature structural material, which shows excellent compressive yield strength at room temperature (1273 MPa) and at the elevated temperature of 900 °C (688 MPa) [16]. Moreover, in our earlier study, the influence of the Cr content, second phase, and sintering temperature on the material properties of WMoVTiCr was investigated [17]. The results indicate that Ti-oxide is a dominant second phase and is considerably dispersed in the BCC solid solution phase [17]. In addition, the high level of Cr encourages the formation of Laves phases, which change the mechanical behavior of the materials [17,18]. It should be also noted that phase stability of RHEAs can be influenced by annealing treatment. An increase in the number of C15 Laves phases can be obtained after the annealing of TiVZrMoW alloy [19]. Thus, in this work, four model RHEAs with a variation of Ti, Ta, and V have been designed and produced by mechanical alloying, namely, WVTaTi, WVTaTiCr, WV_10_Ta_10_TiCr, and WVTaTi_5_Cr. This study aims to understand the effects of alloying elements and mechanical alloying on the formation of second phases, microstructure evolution, and mechanical behavior of the WVTaTiCr model alloys. It is also a critical issue to clarify the effects of oxidation and properties on the model RHEAs as the content of Ti, Ta, and V elements is reduced.

## 2. Materials and Methods

Refractory elemental powders of W, V, Ta, Ti, and Cr with a purity of 99.9% and in the particle size range of 20 μm to 50 μm were used as starting materials to synthesize the model RHEAs by mechanical alloying. In this work, the four WVTaTiCr model RHEAs were studied and named WVTaTi, WVTaTiCr, WV_10_Ta_10_TiCr, and WVTaTi_5_Cr. The chemical composition of the model alloys is shown in Table 1.

The refractory elemental powders were placed in the PM100 planetary ball mill (Retsch, GmbH, Haan, Germany) and the mechanical alloying process was operated at a speed of 300 rpm under an Ar atmosphere. A tungsten carbide medium with a ball-to-powder ratio (BPR) of 10:1 was used during ball milling with different milling durations (4 h, 8 h, and 16 h). The consolidation of the alloyed powders was subjected to a uniaxial pressure of 210 kg/cm^2^ and the green compact samples were further sintered in Ar atmosphere at 1450 °C for 2 h. The heating rate of 2 °C/min was used as the sintering temperature increased from 950 to 1450 °C. and furnace cooling to room temperature. The phase formation of the milled powders and sintered RHEAs was characterized by an X’Pert PRO X-ray diffractometer (XRD, PANalytical, Almelo, The Netherlands) equipped with a copper target. The diffracted intensity of KαCu radiation was measured in the 2θ range from 30° to 110° at a scanning rate of 4° min^−1^. Microstructure development and chemical analysis of the model RHEAs were examined by a Hitachi-4700 scanning electron microscope (SEM, Hitachi High-Tech Corporation, Tokyo, Japan) and energy-dispersive X-ray spectroscopy (EDS, Oxford Instruments, Abingdon, UK). The phase transformation and the crystal structure of different phases were determined using a FEI Tecnai F20 G2 Field Emission Gun TEM (FEI Company, Hillsboro, OR, USA). ImageJ software (V1.53) was used to manually measure the particle size of the model RHEAs. Vickers hardness tests were performed using a load of 9.8 N for a holding time of 15 s at room temperature.

## 3. Results and Discussion

### 3.1. XRD Analysis of Ball-Milled WVTaTiCr Powders

**Figure 1:**
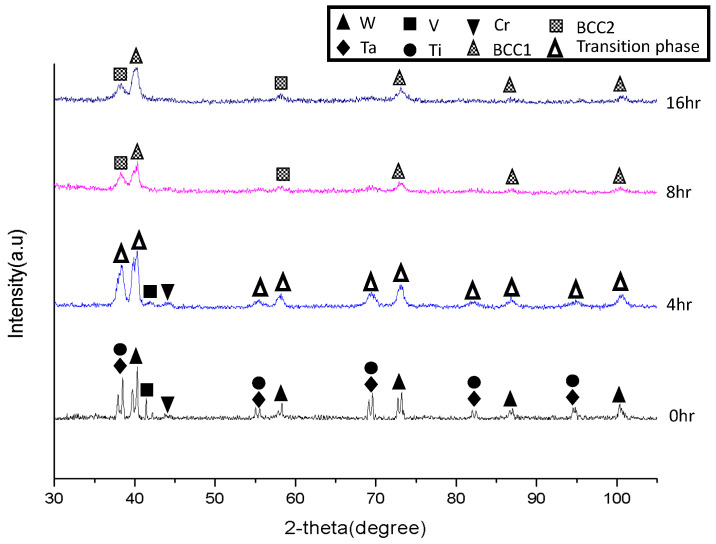
XRD spectra of the WVTaTiCr RHEA powders for different milling times.

The phase formation of the model RHEA powders at different stages of the mechanical alloying process was investigated using XRD. Figure 1 shows the XRD patterns of the WVTaTiCr powders milled for 0 h, 4 h, 8 h, and 16 h. The diffraction peaks of the unmilled powders can be identified easily with the pure metal elements of Ta (PDF#04-0788), W (PDF#04-0806), Ti (PDF#44-1288), V (PDF#65-7446), and Cr (PDF#06-0694). The peak intensities of W were relatively higher than those of the other alloying elements, corresponding to a strong correlation with the atomic number of the constituents. As the milling time increased to 4 h, the intensity of the diffraction peaks decreased significantly and the transition phase was defined in this stage due to metastable states induced by an incomplete solid solution. Moreover, the peak broadening and peak shift appearing on the XRD pattern can be attributed to crystal size refinement, higher lattice strain, and reduced crystallinity, as well as to the formation of a nanocrystalline structure [20]. Additionally, V, Ti, and Cr readily dissolved in the matrix to form a BCC solid solution after 8 h of milling. It should be noted that the alloying sequence of the constituents depends on the melting point of the particular elements [21]. An element with a lower melting point possesses a faster rate of lattice diffusion [21]. In this case, among the constituent elements of the alloy, W and Ta have a higher melting point and thus provide high bonding strength and low self-diffusion, leading to a lower alloying rate. Consequently, after 16 h of milling, the XRD spectrum revealed that the present alloy was composed of two BCC solid solution phases. The major BCC phase is related to W-rich BCC1 and the minor BCC phase corresponds to Ta-rich BCC2. The lattice constants of the BCC1 and BCC2 phases are 3.174 Å and 3.320 Å, respectively.

### 3.2. SEM/EDS Analysis of the Sintered Model RHEAs

#### 3.2.1. WVTaTi Alloys

**Figure 2:**
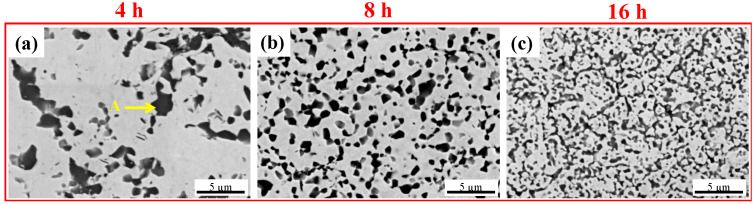
SEM images of the sintered WVTaTi RHEA for different milling times: (**a**) 4 h, (**b**) 8 h, and (**c**) 16 h.

Figure 2 shows the microstructure development of the WVTaTi model RHEAs after different milling times. For the initial stage of milling (4 h) (see Figure 2a), an inhomogeneous microstructure was obtained. The large dark particle with an irregular shape (see point “A”) has a high content of Ti (49.58 at.%) and O (44.29 at.%), as shown by the corresponding EDS analysis (Table 2), suggesting the formation of titanium oxide. This is a common oxide phase that occurs in the Ti-containing HEAs produced by powder metallurgy routes [17,22]. When the milling time was increased to 8 h, as shown in Figure 2b, a relatively uniform microstructure with the dispersion of the dark oxide particles was obtained in the matrix. In the final stage of milling (16 h) (see Figure 2c), a highly refined microstructure with homogeneous particle distribution can be generated through mechanical alloying, which is subjected to high-energy collision with the balls.

#### 3.2.2. WVTaTiCr Alloys

**Figure 3:**
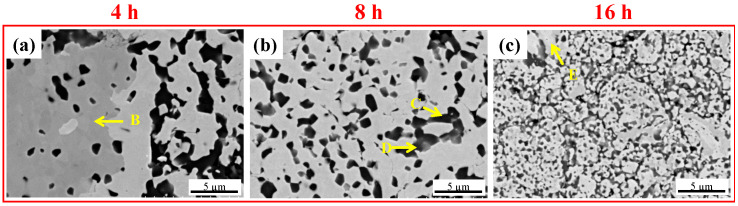
SEM images of the sintered WVTaTiCr RHEA for different milling times: (**a**) 4 h, (**b**) 8 h, and (**c**) 16 h.

The microstructure evolution of the WVTaTiCr alloy during the different milling stages can be seen in Figure 3. It can clearly be observed that the Cr-Ta-enriched phase, which appears as the grey imaged region, was found in the early stage of milling (see point “B” in Figure 3a). It has been reported that RHEAs containing Cr can promote the formation of a Laves phase with AB_2_ stoichiometry such as TaCr_2_, which behaves in a brittle manner at room temperature, acting as a preferred fracture path [23]. In the case of this study, it is worth mentioning that the V element can form a complete solid solution with Cr in the V-Cr-Ta ternary system; therefore, the Laves phase tends to be generated with the chemical composition Ta(V, Cr)_2_ [24]. Consequently, it is believed that the Laves phase in the W-V-Ta-Ti-Cr alloy system may have a composition of (Ti, Ta)(V, Cr)_2_ in the present work.

In addition, in this alloy, the dark particles formed on the matrix correspond to Ti-O or Ta-V-O oxides (see points “C” and “D” in Figure 3b). It should be noted that, in this study, the dark oxide particles have a similar morphology and contrast, which were difficult to distinguish on the SEM images. Additionally, after 16 h of milling, the microstructure was refined and the repeated cold welding and fracturing of the powder particles promoted the precipitation of (Cr, Ta)-rich phases along with dark oxide particles during a long milling time (see Figure 3c). Furthermore, the BCC matrix phase had a bright contrast (see point “E”) containing a higher amount of W, V, and Ta elements. It is worth noting that the depletion of Ti and Cr in the matrix promoted the formation of Tl-rich oxides and Cr-rich phases in this study.

#### 3.2.3. WV_10_Ta_10_TiCr Alloys

**Figure 4:**
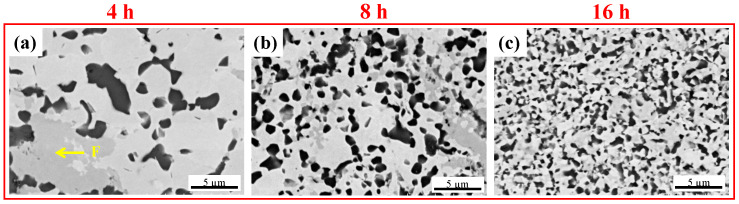
SEM images of the sintered WVTa_10_Ti_10_Cr RHEA for different milling times: (**a**) 4 h, (**b**) 8 h, and (**c**) 16 h.

The SEM images of the WV_10_Ta_10_TiCr model alloys can be seen in Figure 4. In this case, the model alloys with low Ta and V elements limit the formation ability of (Ta, V)-rich oxide particles in the matrix; however, they promote Cr-rich phase formation, which appears as a grey contrast (see point “F”). This phenomenon can be associated with an increase in Cr in this alloy system, which thus promotes Cr-rich phase formation. However, a large number of dark particles corresponding to Ti-rich oxides can also be generated in this sample.

#### 3.2.4. WVTaTi_5_Cr Alloys

**Figure 5:**
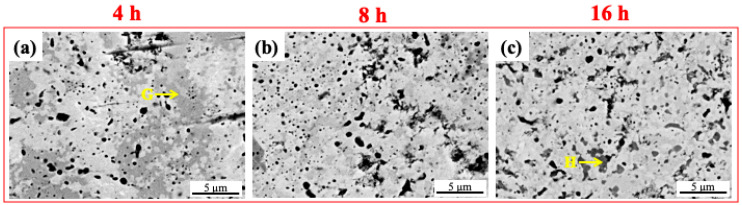
SEM images of the sintered WVTaTi_5_Cr RHEA for different milling times: (**a**) 4 h, (**b**) 8 h, and (**c**) 16 h.

**Figure 6:**
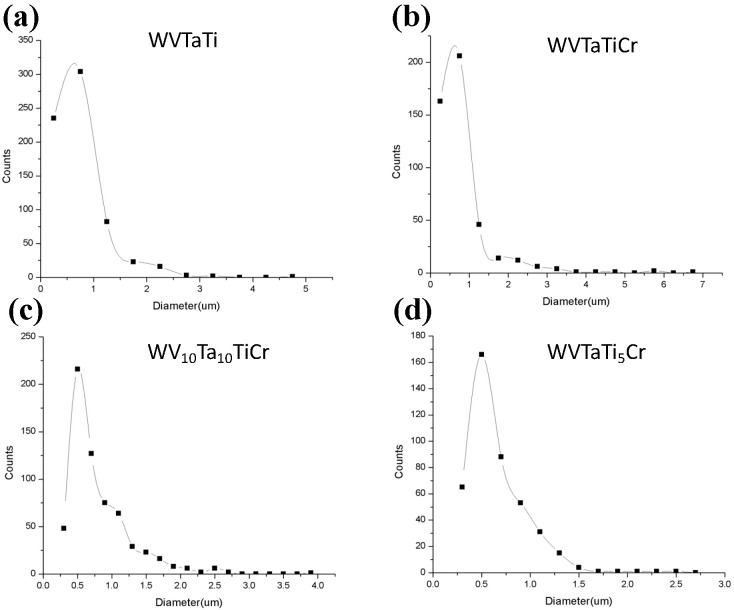
Size distribution of oxide particles in the four model RHEAs: (**a**) WVTaTi, (**b**) WVTaTiCr, (**c**) WV_10_Ta_10_TiCr, and (**d**) WVTaTi_5_Cr.

The microstructure change of the WVTaTi_5_Cr model alloys after different milling times is shown in Figure 5. The low Ti-containing sample demonstrates that there is a significant decrease in a large number of the dark oxide particles formed in the BCC matrix. However, the large region of the (Cr, Ta)-rich phase still can be apparently observed at 4 h of milling (see point “G”) and tends to be refined and of uniform distribution after 8 h of milling, as shown in Figure 5b. Moreover, in a comparison between all the model alloys milled for 16 h (see Figure 2c, Figure 3c, Figure 4c and Figure 5c), it was revealed that the low Ti-containing alloy had a significant effect on microstructure evolution due to the reduction in Ti-rich oxide formation. This can be related to the size distribution of the oxide phase precipitates from the different model alloys, as shown in Figure 6 and Table 3. These results show that, of all the model alloys, the WVTaTi_5_Cr sample has the lowest average particle size (0.66 μm) and volume fraction of the oxide particles (13.22%). It should be pointed out that, although the formation of Ti-rich oxides was encountered in the low level of the Ti model alloy, in this case, the high concentration of Ta and V in the sample promoted the dark particles of Ta-V oxide formation after a long milling time of 16 h (see point “H”).

**Table 2:** EDS analysis corresponding to the model RHEAs (see Figure 2, Figure 3, Figure 4 and Figure 5).

Point (at.%)	W	V	Ta	Ti	Cr	O	Phase
A	1.73	2.73	1.66	49.58	-	44.29	Ti-O
B	6.6	32.32	37.31	0.83	22.93	-	(Cr,V,Ta)-rich
C	4.74	5.75	3.84	39.41	0.48	45.78	Ti-O
D	3.58	29.9	46.8	3.08	0.79	15.86	Ta-V-O
E	31.59	25.69	35.51	1.2	0.45	5.55	BCC matrix
F	8.82	10.08	22.99	7.62	50.49	-	Cr-rich
G	4.41	17.34	40.44	-	30.52	7.28	(Cr,Ta)-rich
H	4.06	54.91	15.89	2.97	5.43	16.75	V-Ta-O

**Table 3:** Average oxide particles size and volume fraction of oxide particles in the four model RHEAs.

Model Alloys	Average Oxide Particles Size (μm)	Vol. Fraction of Oxide Particles (%)
WVTaTi	0.75	30.59
WVTaTiCr	0.82	32.75
WVTa_10_Ti_10_Cr	0.81	32.06
WVTaTi_5_Cr	0.66	13.22

### 3.3. XRD Analysis of the Sintered Model RHEAs

**Figure 7:**
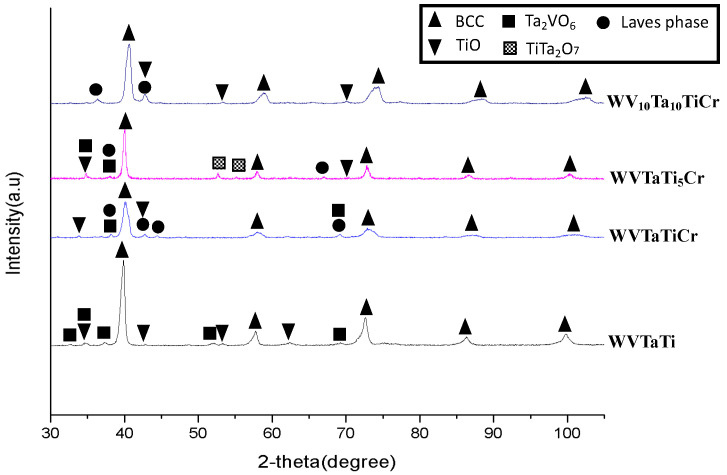
XRD spectra of the sintered model RHEAs.

Figure 7 shows the XRD spectra of the sintered model RHEAs milled for 16 h. The results demonstrate that the four model alloys mainly comprise the major BCC solid solution phase and some other small amounts of different phases. In the WVTaTi sample, the XRD pattern indicates that, apart from the main peaks of the BCC phase, the Ta_2_VO_7_, and Ti-O oxide phases were detected. On the other hand, the Laves phase can be observed in the XRD patterns of the Cr-containing model RHEAs. The Ti-O phase was a dominant second phase in the model RHEAs; however, the phase peaks were weak and could not be clearly observed in the low Ti content sample. In this case, the formability of the Ti-O phase was restricted and replaced by Ta-rich oxides (Ta_2_VO_6_ and TiTa_2_O_7_). It should be pointed out that no Ta-rich BBC2 phase was observed from the XRD patterns of the sintered model alloys, which could be associated with the Ta depletion from the matrix, promoting the formation of Ta-rich oxides or (Cr, Ta)-rich phases.

### 3.4. TEM Investigation of the Sintered Model RHEAs

**Figure 8:**
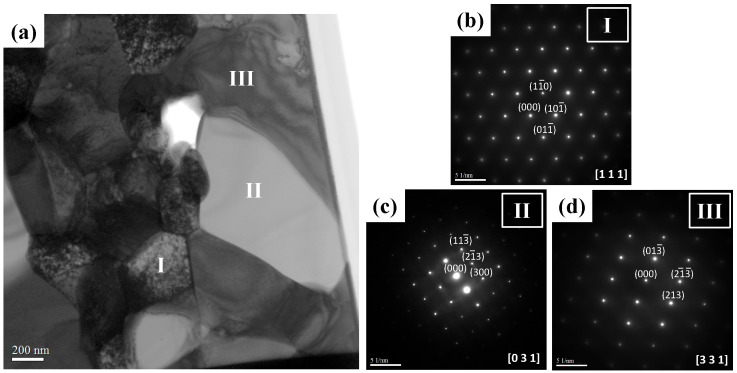
TEM examination of the WVTaTi_5_Cr RHEA: (**a**) the bright file image and corresponding SAD patterns of (**b**) BCC solid solution phase, (**c**) TiTa_2_O_7_, and (**d**) Ta_2_VO_6_ oxide phases.

The phase identification and crystal structure of the WVTaTi_5_Cr RHEA sample after 16 h of milling were further examined by TEM. Figure 8a shows the bright field TEM image of the model alloy with the low Ti content. The matrix phase with the dark contrast (see position “I”) has a high level of W, V, and Ta elements with a composition of 44.86 O-25.05 V-21.78 Ta (at.%). The selected area diffraction pattern of the matrix grain is presented in Figure 8b, indicating a BCC structure along the zone axis of [111], with the lattice parameter calculated as 3.394 Å.

On the other hand, a large grain of ~2 μm with bright contrast (see position ”II”) containing a high concentration of Ta, Ti, and O with a composition of 61.91 O-24.78 Ta-13.15 Ti (at.%) was observed. According to the selected area diffraction (SAD) pattern (see Figure 8c), the Ta-Ti–O phase has been indexed as a TiTa_2_O_7_ oxide, which has a monoclinic crystal structure with lattice parameters a = 20.351 Å, b = 3.801 Å, c = 11.882 Å, and β = 120.19°. It has been reported that TiTa_2_O_7_ can be generated by the conventional high-temperature solid-state reaction method with the mixing of TiO_2_ and Ta_2_O_5_ at the temperature range of 1623 to 1723 K [25]. In addition, at position “III”, the grain with gray contrast has a high content of Ta, V, and O elements. The Ta-V-O oxide particle has been indexed as a Ta_2_VO_6_ tetragonal structure with a *=* 4.750 Å, c = 9.159 Å, along the zone axis of [331] according to the selected area diffraction pattern, as shown in Figure 8d. In this present work, the results imply that in the low Ti-containing model alloy, the Ta element tends to be the second most reactive element, which promotes the formation of Ta-rich oxides during high-temperature sintering. The TEM investigation fits well with the sample analyzed by XRD, as shown in Figure 7.

**Figure 9:**
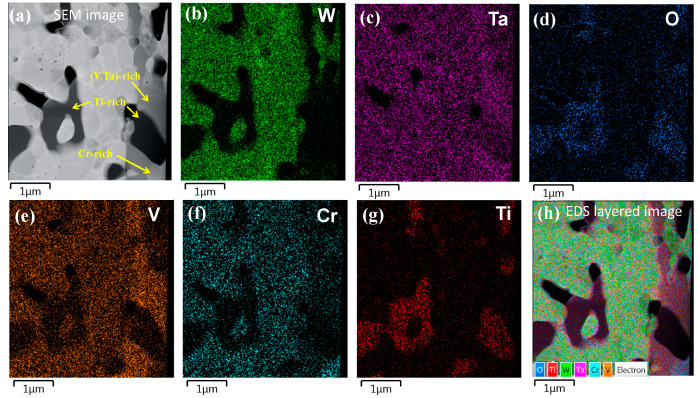
TEM-EDS mapping of the WVTaTi_5_Cr RHEAs: (**a**) SEM image, (**b**) W map, (**c**) Ta map, (**d**) O map, (**e**) V map, (**f**) Cr map, (**g**) Ti map, and (**h**) EDS layered image.

The WVTaTi_5_Cr model alloy was investigated by TEM-EDS mapping, as shown in Figure 9. The results reveal that Ti is the most reactive element in the WVTaTiCr alloy system and promotes TiTa_2_O_7_ oxide formation. Furthermore, it clearly shows that the gray phase is enriched with V, Ta, and O, which corresponds to the Ta_2_VO_6_ phase formation. The Cr-rich phase was also found in the sample, which might be related to the Laves phase (Cr_2_Ta) in this work. It has been reported that the formation of a Laves phase is frequently observed in Cr-containing refractory HEAs [18,26,27].

### 3.5. Hardness of the Sintered Model RHEAs

**Figure 10:**
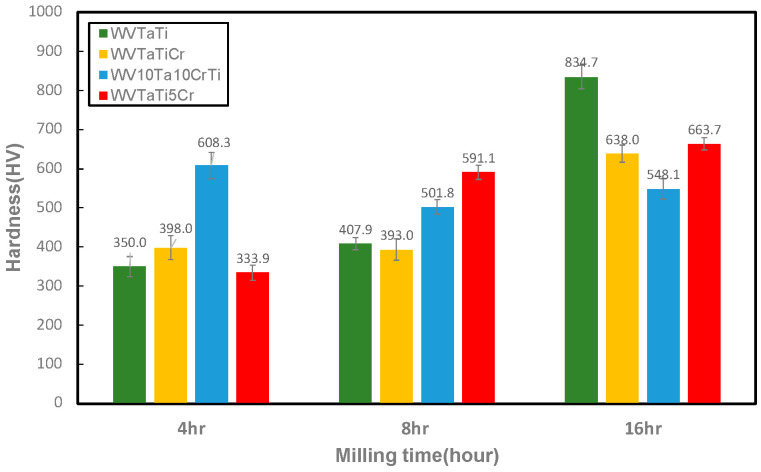
The microhardness of the model RHEAs for the different milling times.

Figure 10 displays the average Vickers microhardness of the four WTaVTiCr RHEAs as a function of milling time. The results indicate that increasing the milling time leads to an increase in the hardness of the model alloys. This suggests that the prolonged milling time provides high strain energy and dislocation density of the milled powders. Therefore, a completed solid solution and refined microstructure in the alloy system can be achieved, improving the hardness of materials. It should be noted that, in the case of the WV_10_Ta_10_TiCr sample, this has a high hardness value in the early stage of milling, which then decreases after a long milling time. This finding could be ascribed to the fact that the WV_10_Ta_10_TiCr alloy has a higher content of W and Cr than the other model alloys, and thus W-rich/Cr-rich phases were generated during the initial milling due to the incomplete milling process and elemental segregation, leading to an increase in the hardness of the model alloy. Moreover, in a comparison of all the model alloys after 16 h of milling, this demonstrates that, of all the model alloys, the WVTaTi alloy has the highest hardness value of 834.7 HV. This suggests that the large number of Ti-rich or Ta-rich oxide particles that formed on the BCC matrix are the primary second phase of the sample. In addition, the WVTaTiCr model alloy has a hardness value of 638 HV, which is a similar result to the literature data for the same alloy system (627.36 HV) [14]. The presence of oxide particles can act as a dispersion strengthening on the materials, thus increasing the hardness of the alloy. Moreover, in the case of the model alloys with Cr, the brittle Laves phase tends to be precipitated at the boundaries of the oxide particles after a long milling time, which can serve as the nucleation sites of micro-cracks and facilitates crack propagation. Accordingly, the oxide particle has a poor bonding strength in the interface and causes delamination with the matrix, resulting in a deterioration in hardness.

## 4. Conclusions

The four WVTaTiCr model RHEAs have been synthesized by mechanical alloying. The influence of alloying elements (Cr, Ta, V, and Ti) and ball milling on the phase formation, oxidation, microstructure, and mechanical properties of the model RHEAs were studied. The results indicate that W-rich BCC1 and Ta-rich BCC2 solid solution phases were obtained after a long milling period. A large number of Ti-O, Ta-Ti-O, and Ta-V-O oxides were generated and dispersed in the BCC matrix of all the model alloys after high-temperature sintering. It should be noted that Ti is the most reactive element to promote the formation of Ti-rich oxides in this work. A complex oxide containing Ti-Ta-O and Ta-V-O has been identified as the TiTa_2_O_7_ and Ta_2_VO_6_ oxide phases by TEM investigation. This reveals that the formation of TiTa_2_O_7_ could be greatened by a solid-state reaction of TiO_2_ and Ta_2_O_5_ subjected to the ball milling process and subsequent high-temperature sintering. Additionally, the increase in the hardness value with increasing the milling time was obtained due to a completed solid solution and refined microstructure achieved after the prolonged milling time. The highest hardness was found in the WVTaTi alloy sample. This suggests that the large number of Ti-rich or Ta-rich oxide particles formed can act as dispersion strengthening on the materials and increase their hardness. Additionally, the (Cr, Ta)-rich phase associated with a Laves phase was observed in the Cr-containing model RHEAs. The brittle Laves phase tends to be formed along with the oxide particle phases and changes the mechanical behavior.

## Figures and Tables

**Table 1:** Chemical composition of the four model RHEAs (at.%).

Model Alloy (at.%)	W	V	Ta	Ti	Cr
WVTaTi	25	25	25	25	-
WVTaTiCr	20	20	20	20	20
WV_10_Ta_10_TiCr	26.66	10	10	26.66	26.66
WVTaTi_5_Cr	23.75	23.75	23.75	5	23.75

## Data Availability

Not applicable.
